# Easily Fabricated Microfluidic Devices Using Permanent Marker Inks for Enzyme Assays

**DOI:** 10.3390/mi7010006

**Published:** 2016-01-12

**Authors:** Coreen Gallibu, Chrisha Gallibu, Ani Avoundjian, Frank A. Gomez

**Affiliations:** Department of Chemistry and Biochemistry, California State University, Los Angeles, 5151 State University Drive, Los Angeles, CA 90032-8202, USA; cgallib@calstatela.edu (C.G.); cgallib2@calstatela.edu (C.G.); aniavoundjian@gmail.com (A.A.)

**Keywords:** microfluidics, glucose oxidase, chromatography paper

## Abstract

In this communication, we describe microfluidic paper analytical devices (μPADs) easily fabricated from commercially available Sharpie ink permanent markers on chromatography paper to colorimetrically detect glucose using glucose oxidase (GOx). Here, solutions of horseradish peroxidase (HRP), GOx, and potassium iodide (KI)were directly spotted onto the center of the μPAD and flowed into samples of glucose that were separately spotted on the μPAD. Using an XY plotter (Roland DGA Corporation, Irvine, CA USA), several ink marks drawn in the paper act as the hydrophobic barriers, thereby, defining the hydrophilic fluid flow paths of the solutions. Two paper devices are described that act as independent assay zones. The glucose assay is based on the enzymatic oxidation of iodide to iodine whereby a color change from clear to brownish-yellow is associated with the presence of glucose. In these experiments, two designs are highlighted that consist of circular paper test regions fabricated for colorimetric and subsequent quantification detection of glucose. The use of permanent markers for paper patterning is inexpensive and rapid and does not require special laboratory equipment or technical skill.

## 1. Introduction

Microfluidics is an exciting technology that has shown considerable promise for producing practical devices, in particular for the analysis of proteins of medical significance. Recent focus has been in the development of point-of-care (POC) diagnostic devices that are inexpensive, simple, disposable, and versatile [1,2,3,4,5]. Lab-on-chip (LOC) technologies can be considered one of the most promising solutions in POC testing due to the ability to miniaturize and integrate many aspects of a laboratory onto a small microfluidic chip. Certain properties of microfluidic technologies including rapid sample processing and precise control of fluids have made them attractive candidates to replace traditional experimental approaches.

In 2007, a new generation of microfluidic paper-based analytical devices (µPADs) was introduced as promising platforms for various applications in resource-limited settings [6]. μPAD technology has shown many advantages including reproducibility, sensitivity, and low limits of detection (LOD). µPADs are created by patterning hydrophobic materials (wax and polymer) in hydrophilic paper. Paper is particularly advantageous for microfluidics due to its ability to wick aqueous fluids without the requirement of active pumping. In addition, paper is thin, available in a variety of thicknesses, is lightweight, easy to stack, store, and transport, is compatible with biological samples given its composition, and is available in many forms with a diverse range of properties [7]. Since the seminal work of Martinez [6,8,9,10,11,12,13,14,15] a myriad of techniques to pattern paper have been detailed including laser [16,17], wax [8,9,10,18,19,20,21], and inject printing [22], plasma etching [23,24], cutting [25], and mechanical plotting [26]. While there have been a number of reports detailing versatile and inexpensive fabrication methods for POC devices, there is an ever-increasing need in resource-limited areas for accessing quality diagnostic testing [27,28,29,30,31,32]. Paper microfluidics have been successfully applied in a number of applications including screening of antibiotics and pharmaceuticals [33], DNA detection [34], chemical screening in multilayer 3D cell cultures [35], enzyme assays [36], multiplex chemical analysis [37], enzyme assays [12], and photoelectrochemical immunoassay [38].

Herein, we describe the design and development of μPADs fabricated from Sharpie ink permanent markers on chromatography paper to colorimetrically detect glucose using glucose oxidase GOx. Using a low-cost XY plotter, several ink marks drawn in the paper act as the hydrophobic barriers that define the hydrophilic fluid flow paths of the solutions. Solutions of reagents and sample are spotted onto the μPADs and the enzymatic oxidation of iodide to iodine is easily visualized and quantified using an inexpensive scanner. The use of permanent markers for paper patterning is a viable alternative to more expensive microfluidic-based patterning techniques for point-of-care (POC) diagnostic devices.

## 2. Experimental Section

### 2.1. Chemicals and Reagents

Horseradish peroxidase (HRP) (E.C. 1.11.1.7), GOx (E.C. 1.1.3.4), and KI were purchased form Sigma Aldrich (St. Louis, MO, USA). Sodium acetate and sodium phosphate were purchased from Fisher Scientific (Pittsburgh, PA, USA).

**Figure 1:**
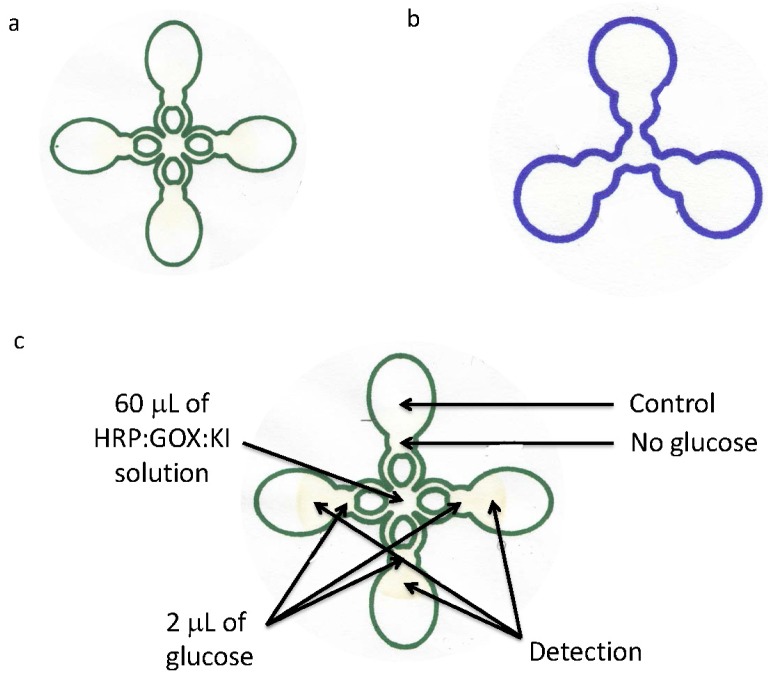
(**a**) Cloverleaf (four-channel) and (**b**) shamrock (three-channel) μPADs used in this study; (**c**) Representation of samples spotted on cloverleaf μPAD.

Briefly, (1) 2.0 μL (50 mM stock) of glucose (0.0, 0.6, 1.9, 2.5, 3.0, 3.3, 4.4, 6.25, 9.20, and 12.1 mM for the cloverleaf chip design) diluted in water was spotted using a micropipette on the circular regions of the μPAD (Figure 1a) and was allowed to dry after spotting; (2) 60 μL of a solution of HRP:GOx:KI was spotted in the middle chip region as shown in Figure 1a. Similar procedures were used with the shamrock chip design as well, spotting the middle chips region with 30 μL of a solution of HRP:GOx:KI instead. Sodium acetate buffer (0.2 M, pH 5.1) was used for the GOx (120 units/mL) solution. Phosphate buffer (0.1 M, pH 6.0) was used for the HRP (30 units/mL) solution. KI (0.6 M) was dissolved in distilled water.

### 2.2. Device Fabrication

Patterns were designed on the computer using Inkscape software, which were subsequently printed on Whatman grade 1 chromatography paper using Sharpie permanent markers and drawn using an XY plotter. Ink from commercially available Sharpie pens generally consist of a hydrophobic resin, solvent, and colorant. The XY plotter creates enough pressure onto the filter cellulose that ink completely penetrates through the chromatography paper. Evaporation of solvent is almost immediate leaving the resin and colorant on the paper resulting in the formation of the hydrophobic walls of the channels. Two chip designs (Figure 1) resembling a cloverleaf (A) (four-channel) and shamrock (B) (three-channel) were printed on areas of 90.3 and 19.7 cm^2^, respectively, containing separate channel regions for sample analysis. In one pattern, the chip consisted of four regions to analyze multiple samples. Here, the center of the chip contained a cocktail of materials. Radiating 1.905 cm from the center of the chip, and in four directions 90° to each other, were located four circular spots. Adjacent to these spots, and co-linear to the center of the chip, is located another region where final analysis of sample is determined. Channels were separated by a distance of 1.6 cm (center of glucose spot circle) and 2.93 cm (center of analyzed circle) from the center of the entire chip. In the shamrock pattern, the cocktail of materials flow 1.24 cm from the center of the chip to the detection spot in the analyzed circle. The three channels are separated 120° from each other, having a distance of 0.84 cm (from the center of the glucose spot circle) and 1.73 cm (from the center of the analyzed circle) from the center of the entire chip.

## 3. Results and Discussion

To demonstrate the efficacy of the Sharpie permanent marker μPADs, we examined the enzymatic oxidation of iodide to iodine. Here, glucose is oxidized by GOx forming hydrogen peroxide that is subsequently reduced to water by HRP concomitant with the oxidation of iodide to iodine. In the present work, solutions of HRP, GOx, and KI were directly spotted onto the center region of the μPAD and flowed into samples of glucose that were separately spotted on the μPAD on the individual circular regions (Figure 1c).

**Figure 2:**
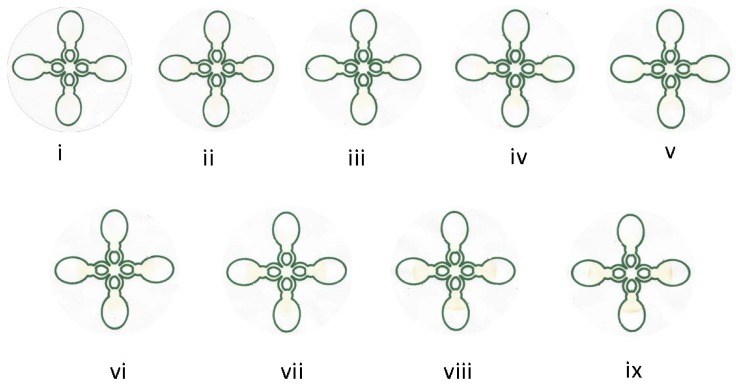
Digital photographic images of the glucose assay for the cloverleaf four-channel μPADs using a range (**i**) 0.6; (**ii**) 1.9; (**iii**) 2.5; (**iv**) 3.0; (**v**) 3.3; (**vi**) 4.4; (**vii**) 6.25; (**viii**) 9.20; and (**ix**) 12.1 mM of concentrations of glucose.

With the present μPAD, samples of different concentrations of glucose were simultaneously analyzed using a cloverleaf–shaped chip (Figure 2). The solution containing GOx:HRP:KI flows across the channels through capillary action and consequently moves onto the region of the chip containing the dried glucose. At this point, oxidation of KI to iodine ccurs resulting in the formation of a colored region in the final chip region. The chip was allowed to dry for ten minutes and the region was scanned on a Canon CanoScan LiDE 210 Desktop Scanner (Canon Inc., Tokyo, Japan) with a resolution of 600 DPI. This data was plotted onto GraphPad Prism 5.0 (GraphPad Software, Inc., La Jolla, CA, USA).

**Figure 3:**
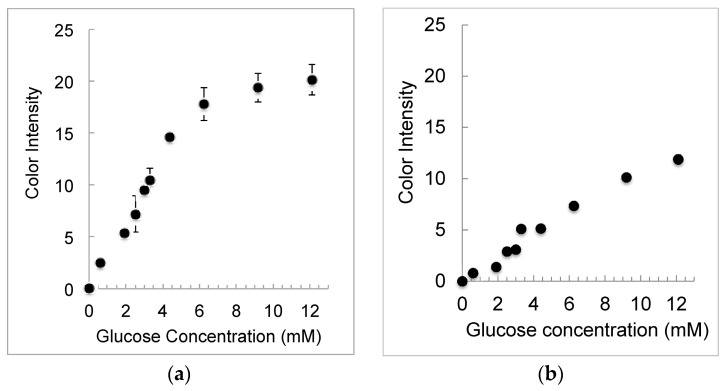
(**a**) Saturation curve of average yellow intensity as a function of glucose concentration for the cloverleaf chip. The error bars represent the relative standard deviation of three independent measurements; (**b**) Saturation curve of average yellow intensity as a function of glucose concentration for the shamrock chip. The error bars represent the relative standard deviation of three independent measurements.

Figure 2 shows the images taken of the detection spot for each channel of the cloverleaf μPAD. We were able to detect differences on the resulting yellow intensity as a function of glucose concentration. As the concentration of glucose increased (0 to 12.1 mM), a noticeable difference in color intensity is observed. Figure 3a is a saturation curve of the corrected average yellow intensity as a function of glucose concentration. The signal for the glucose assay correlates with concentration of analyte. The data and error bars in the figure are the mean and relative standard deviation, respectively. The responses are linear between 0 and 5 mM glucose and deviate from linearity at higher concentrations of analytes before leveling off. For the majority of healthy individuals, normal blood sugar levels range from 4.0 to 6.0 mM when fasting and can be as high as 7.8 mM two hours after eating. Hence, the results show that the μPAD fabricated using permanent markers yields reproducible and accurate quantitative analysis of glucose and within the range of healthy and diabetic patients. The limit of detection (LOD) was approximately 0.3 mM comparable to values (0.5 mM) reported in the literature [8]. While glucose is not usually found in urine, the normal range is 0–0.8 mM. The presence of glucose in the urine is called glycosuria or glucosuria. The μPAD has the capability of measuring multiple samples in parallel simultaneously and in a short period of time. It is also possible to modify the cloverleaf design thereby allowing for the detection and analysis of more than the current three samples.

**Figure 4:**
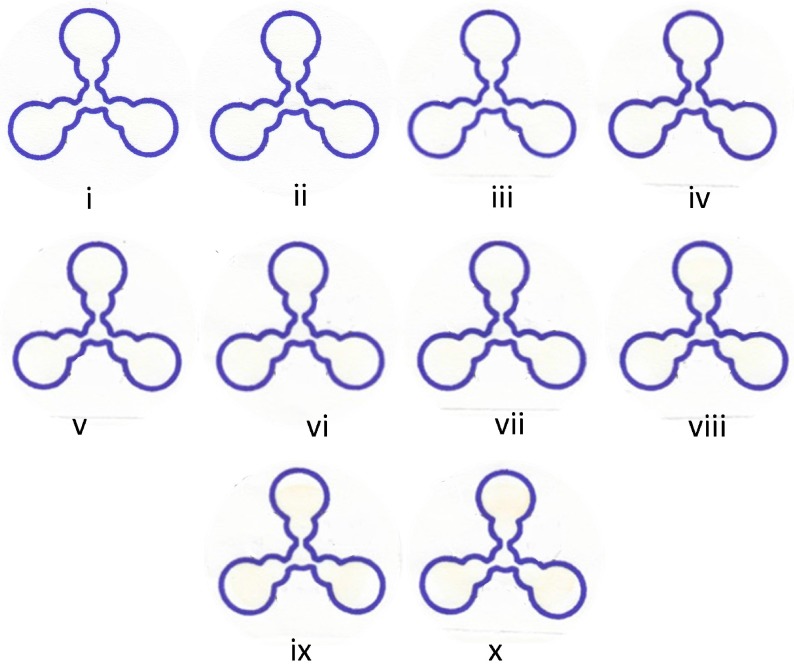
Digital photographic images of the glucose assay for the three-channel μPADs using a range (**i**) 0.0; (**ii**) 0.6; (**iii**) 1.9; (**iv**) 2.5; (**v**) 3.0; (**vi**) 3.3; (**vii**) 4.4; (**viii**) 6.25; (**ix**) 9.20; and (**x**) 12.1 mM of concentrations of glucose.

Figure 4 shows the images taken of the detection spot for each channel of the shamrock-shaped μPADs. Similar results were obtained as that found for the four-channeled chip. Figure 3B is a saturation curve of the corrected average yellow intensity as a function of glucose concentration. It is apparent that the shamrock-shaped μPAD is less sensitive perhaps due to the single channel that connects the sample flows from the center of the μPAD to the three channel outlets. The cloverleaf design has two channels that allow for fluid flow. These results demonstrate that μPADs fabricated using Sharpie permanent markers are reliable and yield quantitative analytical results appropriate for POC testing.

The use of permanent markers allows for facile fabrication of microfluidic platforms, especially in resource limited settings. The devices can easily be produced at low cost in great numbers and in a variety of designs accommodating the analysis of many samples in little time. These reasons make the use of permanent marker inks a logical alternative to other complex fabrication systems including printing and cutting methods.

While the current study utilizes an XY plotter, similar construction of the microfluidic platforms can likely be accomplished using a stencil template. Furthermore, preliminary work using free drawn platforms, without a computer generated design, has shown similar results to those described herein that will make analysis of other relevant chemical species (uric acid, lactic acid, cholesterol, *etc.*) easier and faster.

## 4. Conclusions

We have presented the design and development of a μPAD easily fabricated from commercially available Sharpie ink permanent markers on chromatography paper to detect glucose using the enzyme glucose oxidase. Using an XY plotter, several ink marks drawn on paper act as the hydrophobic barriers to define the hydrophilic fluid flow paths of solutions. Using this method, it is possible to both design and fabricate such devices with new designs within a few hours.

Paper-based microfluidics will continue to emerge as a multiplexable platform for POC diagnostics, thereby transcending the capabilities of existing assays in resource-limited settings. The fabrication simplicity of μPADs using permanent markers should further lower the costs of enzyme assays. This technology holds great promise in bioanalysis due to its sample storage, mixing, and filtration capabilities, sample volume control, and ability to analyze an array of samples simultaneously. Furthermore, their use in other areas including environmental testing, defense-related applications, forensic analysis, and food safety testing holds great promise.
